# Development of a triple antibody sandwich enzyme-linked immunosorbent assay for cassava mosaic disease detection using a monoclonal antibody to *Sri Lankan cassava mosaic virus*

**DOI:** 10.1186/s12985-021-01572-6

**Published:** 2021-05-18

**Authors:** Saengsoon Charoenvilaisiri, Channarong Seepiban, Mallika Kumpoosiri, Sombat Rukpratanporn, Nuchnard Warin, Bencharong Phuangrat, Phakamat Chitchuea, Sirima Siripaitoon, Orawan Chatchawankanphanich, Oraprapai Gajanandana

**Affiliations:** Monoclonal Antibody Production and Application Research Team, National Center for Genetic Engineering and Biotechnology, National Science and Technology Development Agency, 111 Thailand Science Park, Phahonyothin Road, Khlong Nueng, Khlong Luang, Pathum Thani, 12120 Thailand

**Keywords:** Cassava mosaic disease (CMD), Detection, Monoclonal antibody (MAb), *Sri Lankan cassava mosaic virus* (SLCMV), Triple antibody sandwich enzyme-linked immunosorbent assay (TAS-ELISA)

## Abstract

**Background:**

Cassava mosaic disease (CMD) is one of the most devastating viral diseases for cassava production in Africa and Asia. Accurate yet affordable diagnostics are one of the fundamental tools supporting successful CMD management, especially in developing countries. This study aimed to develop an antibody-based immunoassay for the detection of *Sri Lankan cassava mosaic virus* (SLCMV), the only cassava mosaic begomovirus currently causing CMD outbreaks in Southeast Asia (SEA).

**Methods:**

Monoclonal antibodies (MAbs) against the recombinant coat protein of SLCMV were generated using hybridoma technology. MAbs were characterized and used to develop a triple antibody sandwich enzyme-linked immunosorbent assay (TAS-ELISA) for SLCMV detection in cassava leaves and stems. Assay specificity, sensitivity and efficiency for SLCMV detection was investigated and compared to those of a commercial ELISA test kit and PCR, the gold standard.

**Results:**

A TAS-ELISA for SLCMV detection was successfully developed using the newly established MAb 29B3 and an in-house polyclonal antibody (PAb) against begomoviruses, PAb PK. The assay was able to detect SLCMV in leaves, green bark from cassava stem tips, and young leaf sprouts from stem cuttings of SLCMV-infected cassava plants without cross-reactivity to those derived from healthy cassava controls. Sensitivity comparison using serial dilutions of SLCMV-infected cassava sap extracts revealed that the assay was 256-fold more sensitive than a commercial TAS-ELISA kit and 64-fold less sensitive than PCR using previously published SLCMV-specific primers. In terms of DNA content, our assay demonstrated a limit of detection of 2.21 to 4.08 × 10^6^ virus copies as determined by quantitative real-time PCR (qPCR). When applied to field samples (n = 490), the TAS-ELISA showed high accuracy (99.6%), specificity (100%), and sensitivity (98.2%) relative to the results obtained by the reference PCR. SLCMV infecting chaya (*Cnidoscolus aconitifolius*) and coral plant (*Jatropha multifida*) was also reported for the first time in SEA.

**Conclusions:**

Our findings suggest that the TAS-ELISA for SLCMV detection developed in this study can serve as an attractive tool for efficient, inexpensive and high-throughput detection of SLCMV and can be applied to CMD screening of cassava stem cuttings, large-scale surveillance, and screening for resistance.

**Supplementary Information:**

The online version contains supplementary material available at 10.1186/s12985-021-01572-6.

## Background

Cassava (*Manihot esculenta* Crantz, family *Euphorbiaceae*) is one of the important root crops widely grown in Africa, Asia and Latin America [1]. Besides serving as a major staple food in developing countries, it also serves as the raw materials for various starch-based industries in many regions of the world [2]. With an average cassava production of 30 million tons per year, Thailand is the world’s largest exporter of cassava products (e.g. chips, pellets, starch and its derivative products), generating income of approximately USD 3.2 billion per year [3].

Cassava mosaic disease (CMD) caused by a complex of cassava mosaic begomoviruses (CMBs; genus *Begomoviru*s, family *Geminiviridae*) represents one of the major constraints to cassava production in many cassava-growing countries in Africa and Asia [4]. Typical symptoms of CMD include chlorotic mosaic leaves, leaf distortion and deformation, reduced stems, and stunted growth, which can result in yield losses of 20–95% depending on the cultivars and growth environment [5]. Currently, eleven CMB species have been identified, nine in Africa and two in Asia [6].

*Sri Lankan cassava mosaic virus* (SLCMV) and *Indian cassava mosaic virus* (ICMV) are the two CMB species that have been reported in association with CMD in Asia [7, 8]. While ICMV has only been reported in association with CMD in India where it was originally identified, SLCMV (originally identified in Sri Lanka) has also been reported to spread widely and aggressively in India [9, 10] and to have caused the recent CMD outbreaks in several Southeast Asian countries (SEA) including Cambodia [11], Vietnam [12], and Thailand [13]. SLCMV has also recently been reported as the causal agent of CMD observed in cassava germplasm gardens and some cassava production fields in Fujian and Hainan provinces in China [14, 15].

Like other CMBs, SLCMV is transmitted in nature by the whitefly vector *Bemisia tabaci* (Gennadius) and can be disseminated widely from one crop cycle to the next through the use of infected planting materials [16]. Several studies have shown that yield loss from cassava grown from infected stem cuttings is greater than from those infected later by whiteflies (55–77% vs 35–60%) [17, 18]. In term of CMD management, the use of disease-free stem cuttings as planting materials together with regular field monitoring of secondary spread of CMD by whiteflies is considered as an important strategy to reduce yield loss and the incidence of CMD, aside from the development of resistant cassava varieties and the deployment of vector control [19]. Though CMD symptoms can be observed visually, its symptoms can sometimes be difficult to distinguish from those caused by other biotic or abiotic stresses. Towards this end, the availability of an accurate and efficient tool for CMD detection becomes one of the fundamental factors for successful CMD management.

Several molecular and serology-based methods have been employed for the detection of CMBs such as PCR [20], multiplex PCR [21], real-time PCR [22], immunosorbent electromicroscopy [23], double antibody sandwich enzyme-linked immunosorbent assay (DAS-ELISA) [24] and triple antibody sandwich-ELISA (TAS-ELISA) [25]. For SLCMV, methods such as PCR [26–28], multiplex PCR [29, 30], rolling circle amplification (RCA) [12] and plate-trapped antigen-ELISA (PTA-ELISA) [15] have been described. Although molecular-based methods are known to be more sensitive, ELISA is generally more suitable for routine and large-scale CMD screening, due to its simplicity, high throughput and cost-effectiveness. In addition, ELISA requires fewer resources and less technical expertise, and can therefore be adapted for use in most laboratories in developing countries.

This study aimed to develop monoclonal antibodies (MAbs) and a TAS-ELISA for the detection of SLCMV, the causative agent of CMD outbreaks in SEA. MAbs against the recombinant SLCMV coat protein (SLCMV-CP) were prepared using hybridoma technology. The newly prepared MAbs were characterized and used to develop a TAS-ELISA for SLCMV detection in both field-collected cassava leaves and cassava stem cuttings. The sensitivity of the newly developed assay was compared to those of a commercial ELISA kit and PCR. The limit of detection (LOD) of the newly developed TAS-ELISA in terms of DNA content was also determined by quantitative real-time PCR (qPCR). The efficiency of the newly developed assay to detect SLCMV in field samples was assessed in comparison with PCR.

## Materials and methods

### Plant materials

#### For antigen preparation, hybridoma screening and MAb characterization

Cassava plants with CMD symptoms were collected from cassava production fields in Sisaket and Prachin Buri provinces in 2018. SLCMV-infected samples were identified by PCR using previously published SLCMV-specific primers [26] and confirmed by DNA sequence analysis. These SLCMV-infected samples were used as source materials for antigen preparation, hybridoma screening, antibody characterization and TAS-ELISA development. The presence of ICMV in these samples was also ruled out by PCR using ICMV-specific primers. Details of all the primers used in this study are summarized in Additional file 1: Table S1.

#### For specificity analysis

Commercial SLCMV positive control (PC-0424, DSMZ, Germany), SLCMV-infected and healthy cassava plants, as well as plants previously diagnosed for other begomoviruses by PCR and DNA sequencing, including *Ageratum yellow vein virus* (AYVV), *Pepper yellow leaf curl Indonesia virus* (PepYLCIV), *Pepper yellow leaf curl Thailand virus* (PepYLCTHV), *Squash leaf curl China virus* (SLCCNV), *Tomato leaf curl New Delhi virus* (ToLCNDV), *Tomato yellow leaf curl Kanchanaburi virus* (TYLCKaV) and *Tomato yellow leaf curl Thailand virus* (TYLCTHV) were used for specificity analyses of the newly developed MAbs and TAS-ELISA.

#### For assay validation

**Fig. 1:**
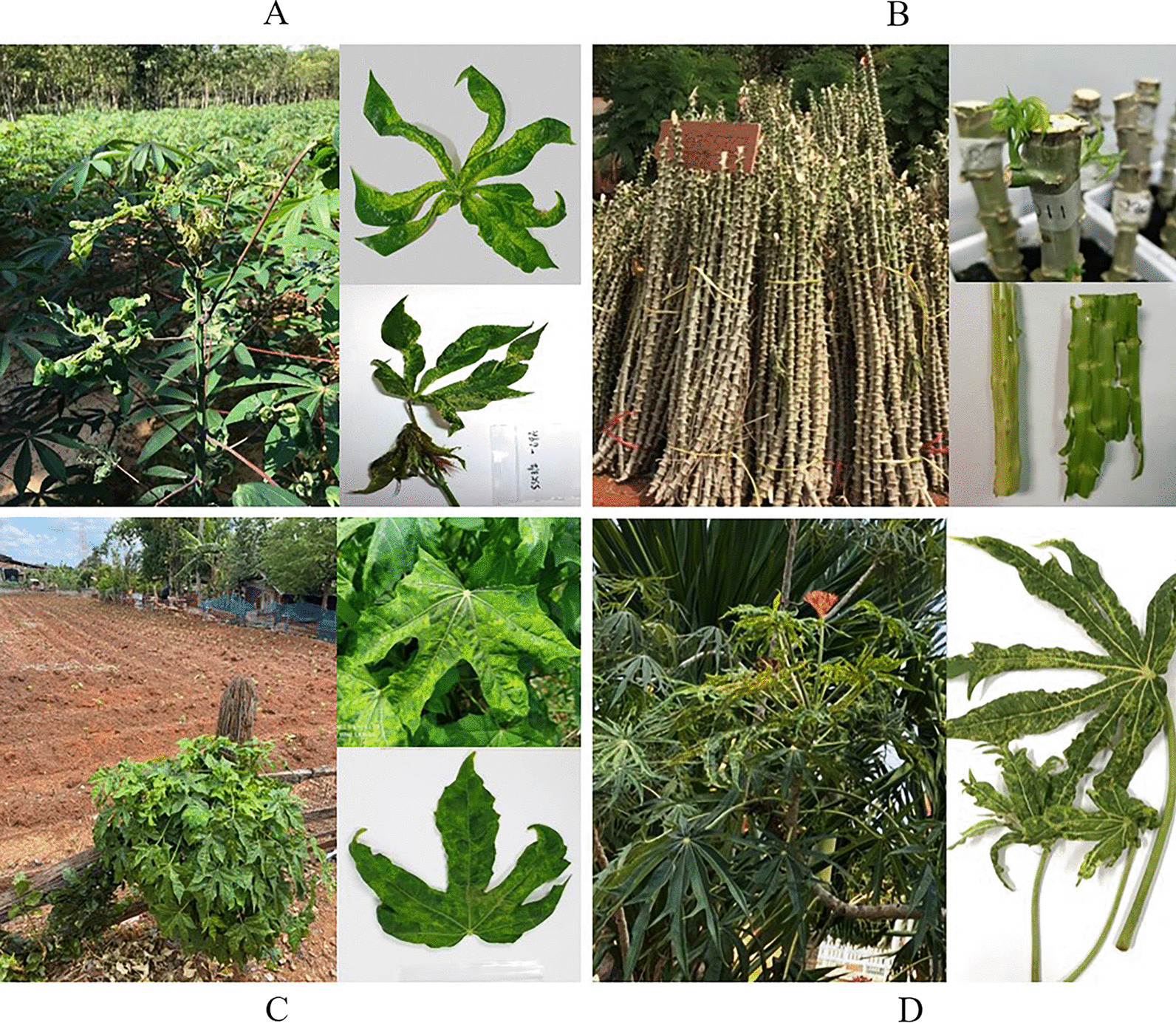
Different types of field samples used in this study. **a** Field-collected cassava leaves. **b** Young leaf sprouts from stem cuttings and green bark from cassava stem tips. **c** Chaya leaves showing CMD-like symptoms. **d** Coral plant leaves showing CMD-like symptoms

Leaves from the top canopy of cassava plants with or without typical CMD symptoms (n = 114) were collected from cassava production fields in Buriram (n = 7), Nakhon Ratchasima (n = 80), Sa Kaeo, and Sisaket (n = 27) provinces between 2018 and 2020. Cassava stems of the shoots that bore symptomatic or asymptomatic leaves in Nakhon Ratchasima province (n = 77) were also collected for testing. Some cassava stems were also purchased from local farmers (n = 275). For each cassava stem, a 30–45 cm long stem cutting was prepared and maintained in soil inside an insect-proof greenhouse to allow sprouting of young leaves (7–14 days). These young leaf sprouts (0.5–2 cm in size) were used as samples for indirect detection of SLCMV in the original cassava stems. Where applicable, green barks from cassava stem tips were also collected and used as samples for direct detection of SLCMV in the stems. Leaves of chaya (*Cnidoscolus aconitifolius*) and a coral plant (*Jatropha multifida*) found bordering CMD-affected cassava fields in Nakhon Ratchasima province were also collected and used for evaluation of the developed TAS-ELISA in comparison with PCR (Fig. 1).

### Cloning and expression of recombinant SLCMV and ICMV coat proteins

The coat protein (CP) gene of SLCMV was amplified from DNA extracts of an SLCMV-infected cassava sample from Sisaket province (SSK3-14) by PCR using primers SLCMV-CPF (5′ GGA TCC ATG TCG AAG CGA CCA GCA 3′) and SLCMV-CPR (5′ AAG CTT TTA ATT GCT CAC TGA ATC 3′). The PCR product was cloned in-frame into the expression vector pQE-80L (QIAGEN, Germany) at the *Hin*dIII and *Bam*H1 restriction sites and transformed into *Escherichia coli* (*E. coli*) DH5α cells. Transformants with the correct insert size were selected by colony PCR using the SLCMV-CPF and SLCMV-CPR primers and grown overnight for plasmid preparation according to the manufacturer’s protocol (QIAPrep Spin Miniprep Kit, Germany). The identity and orientation of the SLCMV-CP gene in the resulting plasmid was confirmed by DNA sequence analysis. The recombinant SLCMV-CP was expressed, purified and analyzed as previously described [31]. Briefly, a bacterial clone containing the correct recombinant plasmid was grown at 37 °C in LB medium containing 100 µg/ml ampicillin to an absorbance at 600 nm (OD_600_) of 0.6. Protein expression was induced by addition of 1 mM isopropyl-1-thio-β-D galactoside (IPTG) for 5 h at 37 °C. Recombinant SLCMV-CP was purified using a Ni–NTA agarose resin column (QIAGEN, Germany) under denaturing conditions following the manufacturer’s recommendations. Purified proteins were analyzed by 12% sodium dodecyl sulfate polyacrylamide gel electrophoresis (SDS-PAGE) and confirmed by western blotting using a commercial MAb to SLCMV (DSMZ, Germany) and MAb D2, which broadly reacts with begomoviruses [31]. The purified SLCMV-CP was dialyzed against phosphate-buffered saline (PBS), pH 7.4, before being used for mouse immunization.

The recombinant CP of ICMV was generated for cross-reactivity testing. ICMV CP ORF was synthesized based on the nucleotide sequence of ICMV isolate Maharashtra (GenBank accession no. AJ314739) and cloned into the pUC57 vector to generate the recombinant plasmid pUC57-ICMV-CP (GenScript, USA). To produce the recombinant ICMV-CP protein, the ICMV-CP gene in pUC57-ICMV-CP was subcloned into an expression vector (pQE-80L, QIAGEN) at the *Hin*dIII and *Bam*HI sites to generate the recombinant expression plasmid pQE-80L-ICMV-CP. The recombinant ICMV-CP protein was expressed and purified as described for SLCMV-CP.

### Preparation of MAbs

The production of MAbs was performed as described previously by our group [31]. Briefly, 6-week-old BALB/c mice were immunized with purified recombinant SLCMV-CP protein by intraperitoneal injection. Sera from immunized mice drawn 7 days after immunization (starting from the third immunization) were tested for antibodies against recombinant SLCMV-CP by PTA-ELISA. After the fifth immunization, the mouse with the highest antibody titer to recombinant SLCMV-CP was boosted with the purified recombinant SLCMV-CP in PBS (pH 7.4) and sacrificed 4 days later for hybridoma preparation. Hybridomas producing antibodies against recombinant SLCMV-CP were screened by PTA-ELISA as previously described. Hybridoma cells found to produce antibodies against recombinant SLCMV-CP with OD_405_ higher than 1.0 were subsequently tested for their reactivity against sap extracts of SLCMV-infected and healthy cassava by western blot analysis as follows. Briefly, leaves of SLCMV-infected or healthy cassava (0.4 g fresh leaf tissue or 0.025 g dried leaf tissue) were homogenized in 1 ml of extraction buffer (0.05 M Tris–HCl; 0.06 M sodium sulfite, pH 8.5). After centrifugation of the samples at 10,000 × *g* for 5 min, supernatants were collected. Two hundred and fifty microliters of protein extracts from SLCMV-infected or healthy plant sap were subjected to SDS-PAGE in one combined well. Separated proteins were electro-transferred to 0.45 µm nitrocellulose membranes (Protran™ GE Healthcare Life Science, Germany) and the blotted membranes were air-dried. Membranes were soaked in Tris-buffered saline plus Tween-20 (TBST; 10 mM Tris pH 8.0; 150 mM NaCl, 0.05% Tween 20) for 10 min and subsequently blocked with 5% (w/v) skim milk in TBST for 1 h. Approximately 3 mm-wide strips were then cut from both sides of the membrane and probed with prefusion sera to locate the position of the virus coat protein band on the membrane. Virus coat protein bands on the membrane (around 31 kDa) were then cut into 2 × 6 mm strips, each of which was transferred to 96-well microtiter plate and incubated with different hybridoma cells previously found to produce antibodies against recombinant SLCMV-CP by PTA-ELISA. After three successive washes with TBST, the membranes were incubated with alkaline phosphatase-conjugated goat anti-mouse polyvalent immunoglobulin (G, A, M) for 1 h. The membranes were washed as described above and incubated with BCIP/NBT substrate solution (Invitrogen, USA) for 5–10 min. Color development was stopped by adding distilled water.

Hybridoma cultures capable of differentiating between SLCMV-infected and healthy cassava were subcultured at limiting dilutions to produce monoclonal cultures of antibody-producing hybridoma cells.

### Characterization of MAbs

The reactivity of MAbs was initially analyzed against recombinant SLCMV-CP, partially purified SLCMV, and sap extract of SLCMV-infected and healthy cassava plants by PTA-ELISA. Specificity of a selected MAb was then determined against sap extracts of SLCMV-infected and healthy cassava as well as sap extracts of plants infected with other begomoviruses by western blot analysis. The isotypes of MAbs were determined using a mouse immunoglobulin isotyping ELISA kit (BD Biosciences, USA). PTA-ELISA and western blot analysis were performed as described previously [31].

### TAS-ELISA for SLCMV detection

#### Plant sap preparation

For field-collected cassava leaf and young leaf sprout samples, sap extracts were prepared by grinding cassava leaf tissues in extraction buffer containing 0.05 M Tris–HCl and 0.06 M sodium sulfite (pH 8.5) at a ratio of 1:5 (w/v, g/mL). For green bark samples, green bark tissues from cassava stem tips were ground in extraction buffer containing 2% PVP, 0.2% Na-DIECA, 0.05 M Tris–HCl, and 0.06 M sodium sulphite (pH 8.5) at a ratio of 1:2.5 (w/v, g/mL). After centrifugation, supernatants were collected for SLCMV detection.

#### TAS-ELISA

TAS-ELISA for SLCMV detection was developed using a previously established rabbit polyclonal antibody against begomovirus (PAb PK) as the capture antibody, and the newly prepared MAb 29B3 as the detection antibody. TAS-ELISA was conducted according to Seepiban et al. [31] with some modifications. Briefly, ELISA plates were coated with PAb PK diluted 1:5000 (v/v) in coating buffer (0.05 M carbonate buffer, pH 9.6) and incubated at 37 °C for 3 h. Plates were washed with phosphate buffered saline plus 0.05% Tween 20 (PBST) and blocked with 2% (w/v) BSA in PBST as previously described. After three washes with PBST, plant sap extracts were added to the wells (in duplicate) and the plates were incubated at 4 °C for overnight. After three washes, MAb 29B3 diluted to 1 µg/ml in 0.5% (w/v) BSA in PBST was added, and the plates were incubated at room temperature for 2 h. After three washes, alkaline phosphatase-conjugated goat anti-mouse IgG (whole molecule) antibody (Sigma-Aldrich, USA) diluted 1:5000 in 0.5% (w/v) BSA in PBST were added, and the plates were incubated at room temperature for 1 h. After the final wash step, p-nitrophenyl phosphate substrate (Life Technologies, USA) diluted in diethanolamine buffer (1 mg/ml) were added, and the plates were incubated at room temperature for 30 min. The absorbance values at 405 nm were measured using a microplate reader (Multiskan FC, Thermo Fisher Scientific, Finland). Reactions were considered positive when the mean absorbance values were at least two-fold higher than that of the healthy control.

### PCR and DNA sequence analysis

Leaf or green bark samples were subjected to DNA extraction using the DNeasy Plant Mini Kit (QIAGEN, Germany) according to the manufacturer’s instruction. SLCMV detection was performed by PCR using SLCMV-specific primers (5′-GAAGGGAGACACATATACCTCG-3′ and 5′-CACATATATATTGTCTCCAATTCAC-3′) targeting the replication initiation protein (*Rep*) gene on SLCMV DNA-A [26]. The PCR products were cloned into the pGEM-T Easy Vector (Promega, USA), and sequenced by 1st BASE Laboratories (Selangor, Malaysia). Nucleotide sequence comparison was performed using the nucleotide BLAST program (http://blast.ncbi.nlm.nih.gov/Blast).

### Preparation of plasmid standard and determination of SLCMV DNA-A genome copy number by quantitative real-time PCR (qPCR)

To quantify the virus copy number, serial tenfold dilutions of a plasmid standard carrying a 2.5-kb SLCMV DNA-A fragment was prepared and used for quantitative analyses by qPCR. Briefly, a 2.5-kb fragment of SLCMV-DNA-A was amplified from DNA extracts of SLCMV-infected plants using primers SLCMV2.5-F: 5′ GCA AGG AAC AGG CTT TAG T 3′ and SLCMV2.5-R: 5′ GGA CTT AAC GCA AAA CCT CT 3′ (targeting the SLCMV DNA-A genome at positions 875-893 and 636-617, respectively). The PCR product was purified and cloned into pGEMT-Easy (Promega, USA) according to the manufacturer’s protocol. The resulting recombinant plasmid was confirmed for its identity by DNA sequence analysis. The plasmid construct harboring the correct SLCMV-DNA-A sequence was linearized by digestion with the *Sal*I restriction enzyme (Thermo Fisher Scientific, USA), purified using the PCR purification kit (QIAGEN, Germany), and quantified using the Qubit 2.0 Fluorometer and the dsDNA BR Assay Kit (Thermo Fisher Scientific, USA). Serial tenfold dilutions of the linearized plasmid was then prepared at copy numbers ranging from 3 × 10^8^ to 30 copies per 5 μl. Conversion from the copy number of interest to the mass of recombinant plasmid was done considering the size of recombinant plasmid (5521 bp), the average molecular weight of one double-stranded DNA molecule (660 g/mole), and the Avogadro’s constant (6.023 × 10^23^ molecule/mole).

To determine the sensitivity of the developed TAS-ELISA in terms of DNA content, serial two-fold dilutions of SLCMV-infected plant extracts were prepared from 3 SLCMV-infected leaf samples and subjected to TAS-ELISA as previously described. For each set of serial dilutions, total DNA was isolated from 100 µl of sap extract from the maximum dilution showing positive results by TAS-ELISA and used as a template for qPCR. Serial ten-fold dilutions of the plasmid standard were also assayed in the same run to generate a standard curve for virus copy number quantification. The qPCR was performed in 20 µl reactions containing 10 µl of 2 × SsoAdvanced™ Universal SYBR® Green Supermix (BIO-RAD, USA), 0.2 µM each of forward and reverse primers (SLCMV-Q4F2377: 5′ GAA TTG CCG ATT GTT TGT GAT TGT G 3′ and SLCMV-Q4R2512: 5′ AGC TTG AGT CTT CCG ACA AAC ATC 3′ newly designed to amplify a 136-bp fragment from the replication initiation protein (Rep) gene on SLCMV DNA-A) and 5 µl of DNA template. The qPCR was carried out on the CFX96 Touch Real-Time PCR Detection System (BIO-RAD, USA) as follows: 1 cycle of 98 °C for 3 min, 40 cycles of 98 °C for 10 s and 60 °C for 30 s. Triplicate reactions were performed for each sample and each standard dilution.

A standard curve plotting the mean Cq values (y-axis) against the log plasmid copy number for each standard dilution (x-axis) was constructed. The equation of the linear regression line, y = m(x) + b, or Cq = slope (log quantity) + y-intercept, was then obtained and used to interpolate the starting copy number of SLCMV DNA-A in test samples. Since only 5 µl of DNA extract (1/10 of the total volume of DNA extract) were used as template for qPCR, all the results were multiplied by ten to determine the copy number of SLCMV DNA-A genomes in sap extracts in the dilution at the TAS-ELISA limit of detection. Quantification of virus copy number by qPCR was considered valid when (1) PCR amplification efficiency was between 90 and 110%, (2) the correlation coefficient was > 0.98, (3) the Cq values of test samples fell within the linear dynamic range of the standards, and (4) No Template Control wells were negative. All data and statistical analyses were done using the CFX Manager Software version 3.1 (BIO-RAD, USA).

### Validation of the newly developed TAS-ELISA for detection of SLCMV in field samples

To validate the newly developed TAS-ELISA for the detection of SLCMV, a total of 490 field samples (483 cassava samples, six chaya samples and one coral plant sample) were tested for SLCMV by the TAS-ELISA and PCR, with the latter serving as the gold standard for this study. The number of positive agreements (PA), positive deviations (PD), negative agreements (NA), and negative deviations (ND) were determined and used to calculate the relative accuracy, relative specificity and relative sensitivity as previously described (relative accuracy = [(PA + NA)/N] × 100%; relative specificity = [NA/(PD + NA)] × 100%; relative sensitivity = [PA/(PA + ND)] × 100%, N = total number of samples [PA + PD + NA + ND]) [32].

## Results

### Expression and purification of recombinant SLCMV-CP

**Fig. 2:**
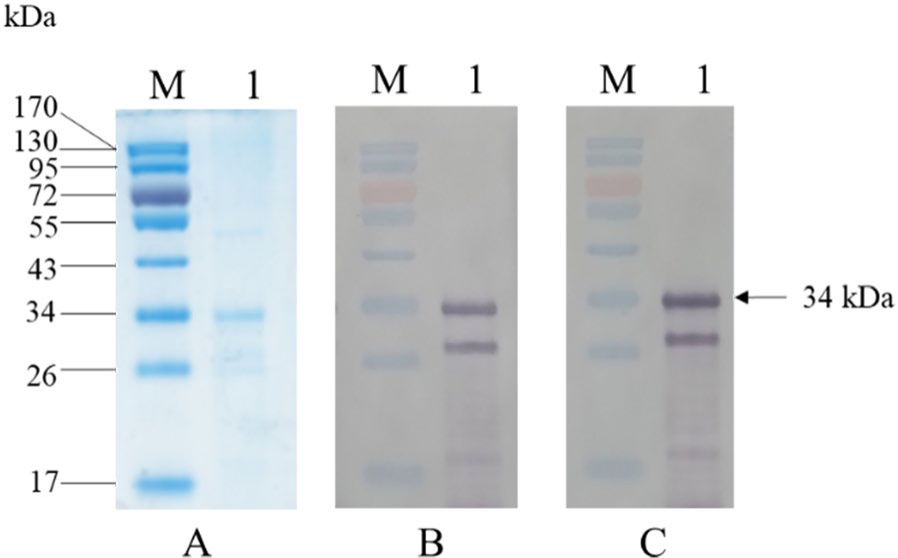
Analyses of recombinant SLCMV-CP protein by SDS-PAGE and western blotting. **a** SDS-PAGE analysis and Coomassie blue staining of purified recombinant SLCMV-CP protein. **b** Western blot analysis of purified recombinant SLCMV-CP protein using a commercial MAb against SLCMV. **c** Western blot analysis of purified recombinant SLCMV-CP protein using MAb D2. Lane M: PageRuler™ Prestained Protein Ladder (Thermo Scientific, USA), Lane 1: purified recombinant SLCMV-CP protein (34 kDa)

The full-length SLCMV-CP gene (771 bp) was successfully amplified from total DNA extracts of SLCMV-infected cassava leaf tissue (SSK3-14) and cloned into the pQE80 expression vector. DNA sequence analysis confirmed the identity and the open reading frame (ORF) of the SLCMV-CP gene in the expression vector (GenBank accession no. MN970012). Recombinant SLCMV-CP was expressed in *E. coli* DH5α cells as a 6xHis-tagged fusion protein (34 kDa) in response to IPTG induction. Recombinant SLCMV-CP was purified using a Ni–NTA agarose resin column under denaturing conditions, yielding a 34-kDa major protein band as detected by 12% SDS-PAGE and Coomassie blue staining (Fig. 2a). The purified recombinant SLCMV-CP was further confirmed by western blot analysis using a commercially available MAb against SLCMV (Fig. 2b) and a previously established MAb against begomovirus, MAb D2 (Fig. 2c). A 34-kDa band visualized in Lane 1 indicated that the recombinant SLCMV-CP protein was successfully expressed and purified. The smaller 28-kDa band observed in Lane 1 is likely to be a degraded form of SLCMV-CP since it was detected by both a commercial MAb to SLCMV and MAb to begomoviruses (MAb D2). The purified SLCMV-CP was then used to immunize BALB/c mice for antibody production.

### Production and characterization of MAbs

After the sixth immunization, a mouse immunized with purified SLCMV-CP was sacrificed for hybridoma preparation. Of 2196 fusion wells, 1076 contained growing hybridomas (48.9%). Among these, 16 showed strong reactivity to the purified SLCMV-CP protein by PTA-ELISA and were capable of differentiating between SLCMV-infected and healthy cassava by western blot analysis (data not shown). After three successive rounds of subcloning, ten stable hybridoma cell lines secreting MAbs to SLCMV-CP were obtained. Of the ten hybridoma clones, MAb 29B3 demonstrated the strongest reactivity to SLCMV-CP by PTA-ELISA and was able to clearly distinguish between SLCMV-infected and healthy cassava by western blot analysis. It was therefore selected for further characterization.

**Fig. 3:**
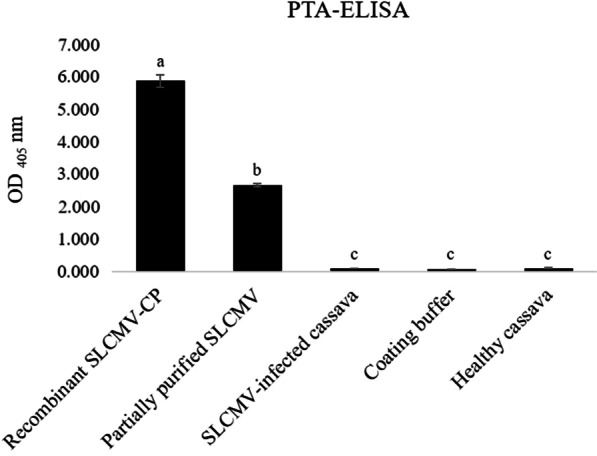
Analysis of MAb 29B3 reactivity by PTA-ELISA. Reactivity of MAb 29B3 was analyzed against recombinant SLCMV-CP, partially purified SLCMV and SLCMV-infected cassava by PTA-ELISA. The OD_405_ values indicate the mean values obtained from duplicate wells. Different letters on each column indicate a significant difference compared to other column at *p* ≤ 0.01 based on Tukey’s HSD test (SPSS software version 11.5 for Window®). The cut-off value was twice the mean OD_405_ of the negative controls (Coating buffer for purified recombinant SLCMV-CP and partially purified virus; sap extract of healthy cassava for SLCMV-infected cassava)

**Fig. 4:**
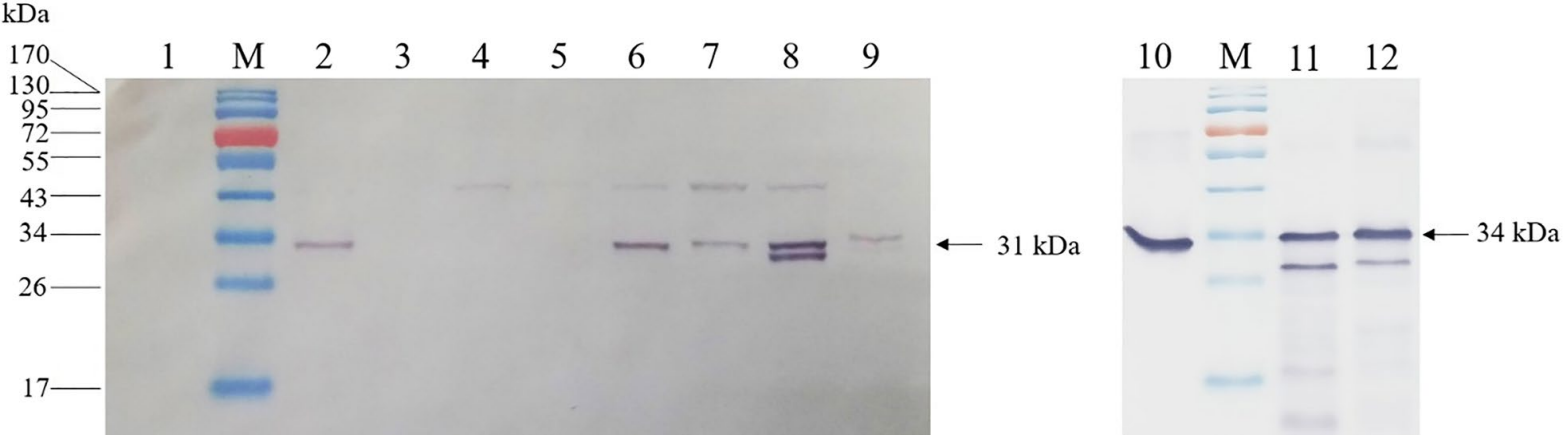
Specificity analysis of MAb 29B3 against SLCMV and select begomoviruses by western blot analysis. Protein lysates prepared from sap extracts of healthy cassava or sap extracts of plants infected with SLCMV and different types of begomoviruses, partially purified SLCMV, recombinant SLCMV-CP and ICMV-CP protein were subjected to western blotting and probing with MAb 29B3. Lane 1: healthy cassava, Lane 2: SLCMV-infected cassava, Lane 3: AYVV-infected *Ageratum conyzoides*, Lane 4: PepYLCIV-infected pepper, Lane 5: TYLCKaV-infected eggplant, Lane 6: SLCCNV-infected pumpkin, Lane 7: ToLCNDV-infected cucurbits, Lane 8: PepYLCTHV-infected pepper, Lane 9: TYLCTHV-infected tomato, Lane 10: partially purified SLCMV, Lane 11: recombinant SLCMV-CP, Lane 12: recombinant ICMV-CP, Lane M: PageRuler™ Prestained Protein Ladder (Thermo Scientific, USA). Each lane was loaded with 5 μl of protein extracts from plant sap, 10 μg of a partially purified virus or 0.1 μg of recombinant coat protein. The size of the coat protein in plant extract and partially purified virus (31 kDa) and the recombinant coat protein (34 kDa) are indicated

The isotype and subclass of MAb 29B3 was determined to be IgG1 with a kappa light chain. Initial characterization by PTA-ELISA showed that while MAb 29B3 reacted strongly with the recombinant SLCMV-CP protein and partially purified SLCMV, it failed to detect SLCMV in sap extracts of SLCMV-infected cassava leaf tissues by this platform (Fig. 3). Specificity analysis of MAb 29B3 by western blot analysis showed that MAb 29B3 reacted strongly with the 31-kDa SLCMV-CP band in protein extracts from SLCMV-infected cassava. No cross-reactivity was observed with those from the healthy cassava control (Fig. 4). The 31-kDa bands were also observed for protein extracts from plants infected with SLCCNV, ToLCNDV, PepYLCTHV, and TYLCTHV, but not for those infected with AYVV, PepYLCIV, or TYLCKaV. The cross-reactivity to some begomoviruses was not unexpected owing to high CP amino acid sequence similarities among begomoviruses. Phylogenetic analysis of the deduced CP amino acid sequences revealed that SLCMV-CP shared 78–91% identity with that of PepYLCTHV, TYLCTHV, SLCCNV and ToLCNDV (MAb 29B3-positive begomoviruses), while sharing 73–81% identity with that of AYVV, PepYLCIV and TYLCKaV (MAb 29B3-negative begomoviruses) (data not shown). Given that SLCCNV, ToLCNDV, PepYLCTHV, and TYLCTHV are begomoviruses that have only been reported in tomatoes, peppers or cucurbits, the use of MAb 29B3 for the development of TAS-ELISA for SLCMV detection in cassava plants should not be a problem.

Nevertheless, it should be noted that the CP amino acid sequence of ICMV, one of the two cassava mosaic viruses reported in Asia, also shares a very high sequence identity (96–100%) with SLCMV- CP, and thus has the potential to be detected with MAb 29B3 produced in this study. However, since ICMV infecting cassava has not been reported in Thailand to date, the recombinant CP of ICMV was generated by subcloning the synthesized ICMV CP ORF into an expression vector and used for cross-reactivity testing. As seen in Fig. 4, cross-reactivity was observed between MAb 29B3 and the recombinant ICMV-CP.

### Development of TAS-ELISA for SLCMV detection

A TAS-ELISA for SLCMV detection in cassava leaves and stem cuttings was successfully developed using PAb PK, a PAb against begomovirus, as the capture Ab and the newly established MAb 29B3 as the detection Ab. Two different extraction buffers were also optimized for efficient sap extract preparation from both cassava leaf and green bark tissues (see Materials and methods).

**Fig. 5:**
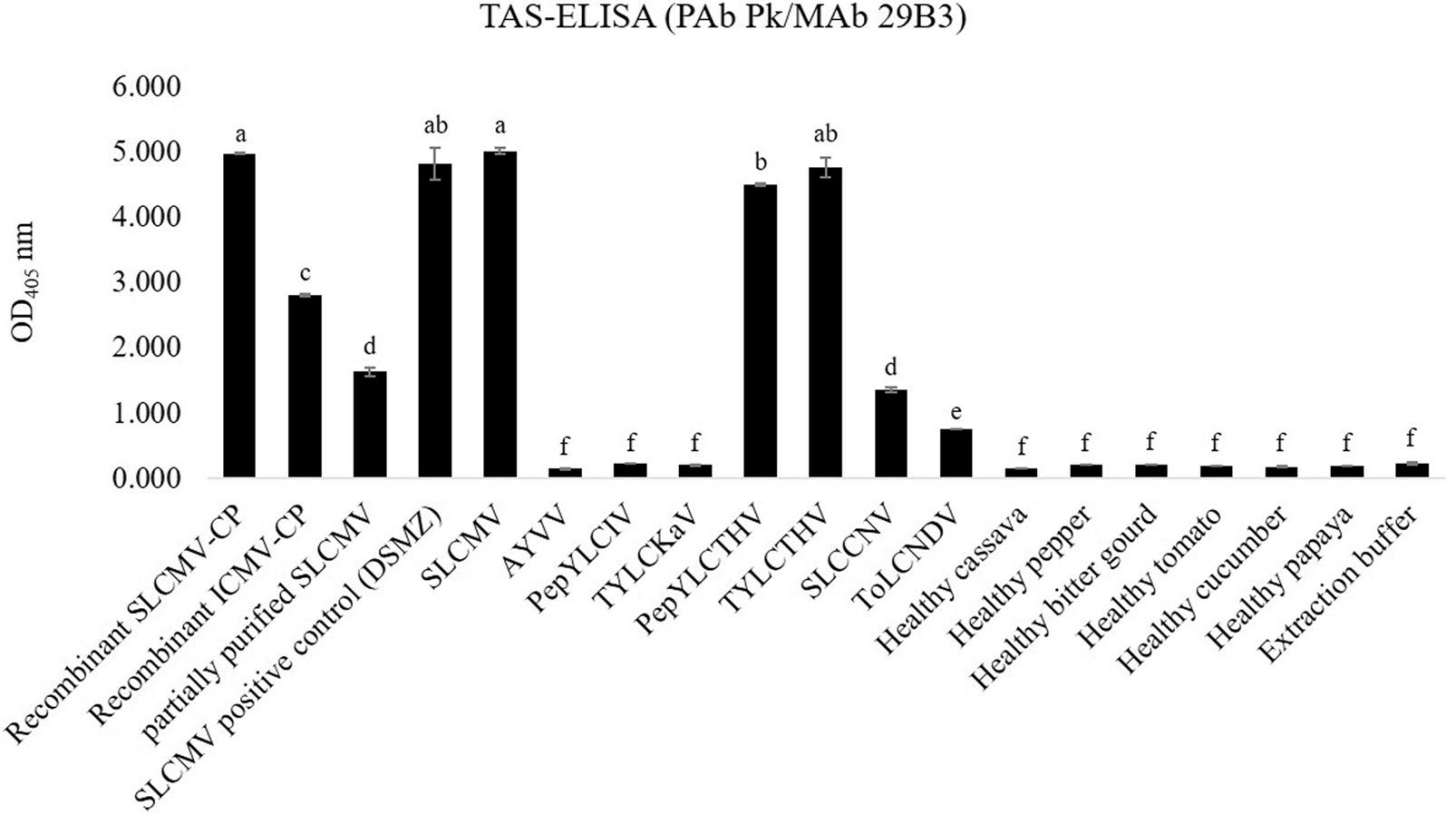
Specificity analysis of TAS-ELISA using PAb PK and MAb 29B3. The specificity of the developed TAS-ELISA was investigated against the recombinant SLCMV-CP and ICMV-CP protein, partially purified SLCMV, a commercially available SLCMV positive control, and sap extracts from plants infected with SLCMV as well as other begomoviruses. The OD_405_ values indicate the mean values obtained from duplicate wells. Different letters on each column indicate a significant difference compared to other column at *p* ≤ 0.01 based on Tukey’s HSD test (SPSS software version 11.5 for Window®). The cut-off value was twice the mean OD_405_ of the negative controls (extraction buffer for purified recombinant CP and partially purified virus; sap extract of healthy plants for virus-infected plants)

The specificity of the developed TAS-ELISA was investigated against the recombinant SLCMV-CP and ICMV-CP proteins, partially purified SLCMV, a commercially available SLCMV positive control, and sap extracts from plants infected with SLCMV as well as some other begomoviruses available in our laboratory. As shown in Fig. 5, MAb 29B3 reacted strongly with the recombinant SLCMV-CP and ICMV-CP proteins, partially purified SLCMV, and the SLCMV positive control. Unlike the PTA-ELISA platform, the newly developed TAS-ELISA was able to clearly differentiate between sap extracts from SLCMV-infected samples and the healthy cassava control (OD_405_ = 5.009 ± 0.047 vs OD_405_ = 0.161 ± 0.003). Specificity tests against other begomoviruses showed cross-reactivity in sap extracts of plants infected with PepYLCTHV, TYLCTHV, SLCCNV and ToLCNDV. No cross-reactivity was observed with AYVV-, PepYLCIV-, and TYLCKaV- infected plant samples or healthy plant controls. As in western blot analysis, cross-reactivity was also observed with the recombinant ICMV-CP, suggesting that the newly developed TAS-ELISA could also be used for ICMV screening.

### Sensitivity comparison

To investigate the sensitivity of the newly developed TAS-ELISA, an SLCMV-infected leaf sample was used to prepare sap extracts as described above. Serial two-fold dilutions of SLCMV-infected extracts were then prepared in sap extracts of healthy cassava leaf tissues from 1/160 to 1/167,772,160 (w/v, g/mL) and subjected to SLCMV detection by our newly developed TAS-ELISA, a commercial SLCMV-TAS-ELISA kit (DSMZ, Germany), and by PCR using previously published SLCMV-specific primers [26]. For ELISA, 100 µl of each sap dilution were analyzed in duplicate wells. For PCR, total DNA was isolated from 100 µl of each sap dilution and used as the template for SLCMV detection.

**Fig. 6:**
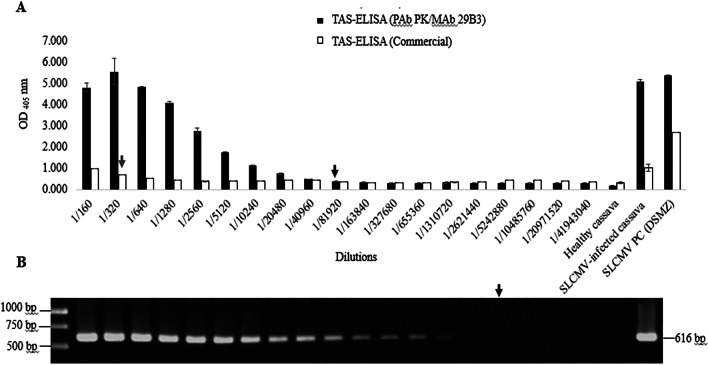
Comparison of SLCMV detection sensitivity between the TAS-ELISA developed in this study, a commercial TAS-ELISA, and PCR. Sap extracts from SLCMV-infected cassava were prepared by serial two-fold dilutions in sap extracts of healthy cassava and subjected to SLCMV detection by TAS-ELISA and PCR. **a** SLCMV detection by the newly developed TAS-ELISA (black bar) and a commercial TAS-ELISA kit (white bar). OD_405_ values indicate the mean values obtained from duplicate wells. The cut-off value was twice the mean OD_405_ of the negative control (sap extract of healthy cassava). **b** SLCMV detection by PCR using SLCMV-specific primers [26]. Arrows indicate the highest dilution that gave a positive result for each method

As shown in Fig. 6, the newly developed TAS-ELISA was able to detect SLCMV in infected cassava sap extracts diluted up to 1:81,920, while the commercial TAS-ELISA kit was able to detect SLCMV in infected cassava sap extracts diluted up to 1:320. It should be noted that the sample used for serial dilution preparation (PRJ-44) was already confirmed by PCR as positive for SLCMV and negative for ICMV infection (Additional file 2: Fig S1), therefore the reactivity observed in this experiment should solely be from the reaction between MAb 29B3 and SLCMV. PCR using previously published SLCMV-specific primers was found to detect SLCMV in infected cassava extracts diluted at 1:5,242,880. These results indicated that the newly developed TAS-ELISA based on PAb PK and MAb 29B3 can be considered a highly sensitive method for SLCMV detection in infected cassava leaf samples, with a detection limit of 256 times greater than that of a commercial ELISA kit, and 64 times less than that of PCR.

### Limit of detection analysis

**Fig. 7:**
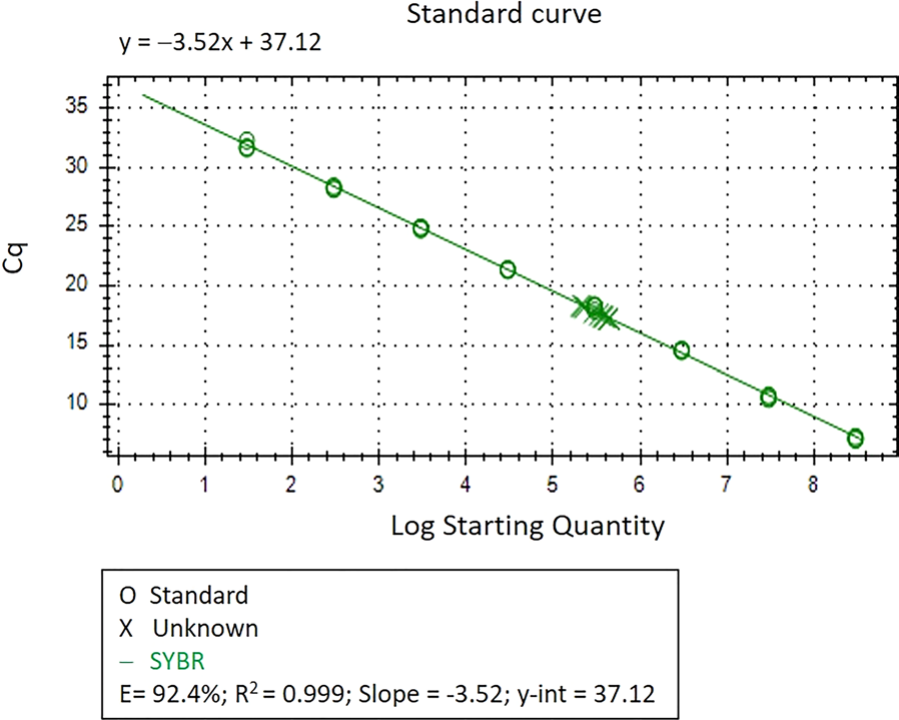
SLCMV genome copy number quantification by qPCR. A serial tenfold dilution of the plasmid standard (pGEM-T Easy vector containing a 2.5-kb fragment of SLCMV DNA-A) ranging from 3.0 × 10^8^ to 30 copies per 5 µl was prepared and used for quantitative analyses by qPCR. A linear regression line with a PCR amplification efficiency of 92.4% and a correlation coefficient of 0.999 was obtained and used to determine the copy number of SLCMV-DNA-A in the test samples. All samples were assayed in triplicate. Data and statistical analyses were done using the CFX Manager Software version 3.1 (BIO-RAD, USA). Circle “ο” indicates Cq from each replicates of standard plasmid, cross mark “×” indicates Cq from each replicates of three test samples

To determine the sensitivity or limit of detection (LOD) of the newly developed TAS-ELISA in terms of DNA content, three sets of serial two-fold dilutions of SLCMV-infected plant saps were prepared and subjected to SLCMV detection by TAS-ELISA in three independent experiments. The same amount of sap extract used for SLCMV detection by ELISA (100 µl) was also taken for DNA isolation and subjected to qPCR to determine SLCMV DNA genome copy numbers. A standard curve for the copy number quantitation was generated using serial ten-fold dilutions ranging from 30 to 3 × 10^8^ copies of a recombinant plasmid harboring a 2.5-kb fragment of SLCMV DNA-A. As shown in Fig. 7, the standard curve generated revealed a linear regression line with PCR amplification efficiency of 92.4% and a correlation coefficient of 0.999, and thus can be used to interpolate the virus copy number in sap extracts. Cq values obtained from test samples all fell within the dynamic range of the standard curve. Based on their mean Cq values, SLCMV DNA copy numbers in the maximum dilution of sap extracts tested positive by TAS-ELISA were estimated to range between 2.21 × 10^6^ to 4.08 × 10^6^ copies (Table 1).

**Table 1:** Determination of SLCMV genome copy number in SLCMV-infected sap extracts

Sample type	Sample name (known copy no./5 µl)	Cq values^b^ Mean ± SD (n = 3)	Calculated copy no./5 µl template^b^ Mean ± SD (n = 3)	Calculated copy no./ELISA well^c^ Mean ± SD (n = 3)
Plasmid standard with known copy number	STD 1 (3.00E+08)	7.19 ± 0.12		
	STD 2 (3.00E+07)	10.69 ± 0.15		
	STD 3 (3.00E+06)	14.59 ± 0.06		
	STD 4 (3.00E+05)	18.22 ± 0.23		
	STD 5 (3.00E+04)	21.42 ± 0.04		
	STD 6 (3.00E+03)	24.93 ± 0.09		
	STD 7 (3.00E+02)	28.33 ± 0.13		
	STD 8 (30)	31.94 ± 0.35		
Maximum dilution of sap extract tested positive by TAS-ELISA^a^	Sample A dilution A7^a^	17.44 ± 0.22	4.08E+05 ± 5.68E+04	4.08E+06 ± 5.68E+05
	Sample B dilution B6^a^	18.38 ± 0.08	2.21E+05 ± 1.19E+04	2.21E+06 ± 1.19E+05
	Sample C dilution C5^a^	17.66 ± 0.23	3.55E+05 ± 5.17E+04	3.55E+06 ± 5.17E+05

^a^Three sets of serial two-fold dilutions of SLCMV-infected plant saps were prepared from 3 infected samples and subjected to SLCMV detection by TAS-ELISA in three independent experiments. The same amount of sap extract from the maximum dilution tested positive by TAS-ELISA was used for DNA isolation and subjected to qPCR to determine the SLCMV DNA genome copy number. A7, B6 and C5 indicate the maximum dilution obtained from serial dilution sets A, B and C, respectively

^b^Data and statistical analyses were done using the CFX Manager Software version 3.1 (BIO-RAD, USA). Copy numbers were calculated from the equation y =  − 3.52x + 37.12, which was obtained from the linear regression line shown in Fig. 7

^c^Since only 5 µl of DNA extract (1/10 of the total volume of DNA extract) were used as template for qPCR, all the results were multiplied by ten to determine the copy number of SLCMV DNA-A genomes in the maximum dilution of sap extract tested positive by TAS-ELISA^a^

### Validation of the newly developed TAS-ELISA for SLCMV detection in field-collected cassava samples

To validate the newly developed TAS-ELISA for SLCMV detection, the assay was applied to detect SLCMV in cassava leaves and stems collected from cassava production fields or purchased from local farmers in Buriram, Nakhon Ratchasima Prachin Buri, Sa Kaeo, and Sisaket provinces between 2018 and 2020. For cassava stems, green barks from cassava stem tips (where applicable) and young leaf sprouts germinating from stem cuttings after being maintained in a greenhouse for 7–14 days were used as samples for direct and indirect SLCMV detection in the original cassava stems. The results obtained by TAS-ELISA were then compared to those obtained by PCR, which served as a standard method for SLCMV detection in this study. A representative image of SLCMV detection in field samples by PCR is shown in Additional file 3: Fig S2.

For field-collected cassava leaf samples (n = 114), results showed that all 57 symptomatic leaf samples tested positive by both TAS-ELISA and PCR. Interestingly, two of 57 asymptomatic leaf samples also tested positive by TAS-ELISA, while four tested positive by PCR (Table 2). For field-collected cassava stems (n = 77), all 35 leaf sprouts from stem cuttings of symptomatic cassava plants tested positive by both TAS-ELISA and PCR. In addition, four of 42 leaf sprouts from stem cuttings of asymptomatic cassava plants were also tested positive by both TAS-ELISA and PCR. None of the 275 young leaf sprouts from cassava cuttings purchased from local farmers tested positive by either TAS-ELISA or PCR. For green bark samples (n = 17), all five green bark samples obtained from the tip of cassava stems of symptomatic cassava plants tested positive by TAS-ELISA and PCR, while one of 12 green bark samples obtained from asymptomatic cassava plants tested positive by both TAS-ELISA and PCR. Besides cassava plants, chaya and coral plant samples found at the border of CMD-affected cassava fields in Nakhon Ratchasima province were also tested for SLCMV. Results showed that all three chaya and one coral plant samples with CMD-like symptoms tested positive by both TAS-ELISA and PCR, whereas those without CMD symptoms (n = 3) tested negative by both methods. BLASTn analysis of partial Rep gene sequences derived from chaya and coral plants in this study (GenBank accession numbers MT921843 and MT921844, respectively) revealed 98.4–100% nucleotide sequence identities (NSI) with various SLCMV isolates previously reported on GenBank, thus confirming the detection of SLCMV infection in chaya and coral plant in this study.

**Table 2:** Comparison of SLCMV detection in different types of field samples by TAS-ELISA and PCR

Sample types	SLCMV detection No.positive/no.tested	
	TAS-ELISA	PCR
*Field-collected cassava leaf samples*		
Leaves with CMD symptoms	57/57	57/57
Leaves without CMD symptoms	2/57	4/57
Total	59/114	61/114
*Young leaf sprouts from cassava stem cuttings*		
Stem cuttings from cassava with CMD symptoms	35/35	35/35
Stem cuttings from cassava without CMD symptoms	4/42	4/42
Stem cuttings randomly purchased from local farmers	0/275	0/275
Total	39/352	39/352
*Green bark at the tip of cassava stems*		
Stem cuttings from cassava with CMD symptoms	5/5	5/5
Stem cuttings from cassava without CMD symptoms	1/12	1/12
Total	6/17	6/17
*Other plants*		
Chaya leaves with CMD-like symptoms	3/3	3/3
Chaya leaves without CMD-like symptoms	0/3	0/3
Coral plant leaves with CMD-like symptoms	1/1	1/1
Total	4/7	4/7
Total samples	108/490	110/490

Taken together, our results suggest that the newly developed TAS-ELISA is sensitive and accurate for SLCMV detection in field-collected cassava samples with 99.6% relative accuracy, 98.2% relative sensitivity and 100% relative specificity as compared to PCR (See Additional file 4: Table S2 for the calculation of the relative specificity, relative sensitivity and relative accuracy of the TAS-ELISA as compared to the PCR).

## Discussion

Cassava growers in South and Southeast Asia account for 30% of the world’s cassava production, with Thailand ranking as the world’s largest exporter of cassava products and the world’s second largest cassava producer after Nigeria [1]. The impact of recent outbreaks of SLCMV in SEA on cassava-based industries has undoubtedly become regional and global concerns.

For developing countries, an accurate and yet affordable diagnostic method is one of the essential tools for epidemiological study and disease management. ELISA-based methods are considered some of the most convenient, high-throughput and cost-effective tools, which can be applied in conjunction with other methods such as PCR for epidemiological studies, CMD screening of cassava stem cuttings, and breeding work for SLCMV-resistant varieties.

In this study, MAbs against recombinant coat protein of SLCMV were produced and a TAS-ELISA based on the newly established MAb 29B3 and an in-house PAb against begomovirus was developed for SLCMV detection in three different sources of cassava tissues including field-collected cassava leaves, young leaf sprouts budding from stem cuttings, and green barks from cassava stem tips. SLCMV coat protein was chosen as the target antigen for SLCMV detection in this study based on its high amino acid sequence conservation among different isolates and the fact that it is the only structural protein that forms the shell of SLCMV particles and also the most abundant viral protein in infected plant tissue during infection [35, 36]

Initial characterization by western blotting showed that MAb 29B3 could react with recombinant SLCMV-CP, partially purified SLCMV, sap extracts from SLCMV-infected cassava leaf tissues without any cross-reactivity with the healthy cassava control. Specificity tests against other begomoviruses commonly found in tomatoes, peppers and cucurbits crops in Thailand showed that MAb 29B3 could also react with PepYLCTHV, TYLCTHV, SLCCNV, and ToLCNDV, but not with AYVV, PepYLCIV and TYLCKaV. Nevertheless, given that these begomoviruses have never been reported in cassava to date, the cross-reactivity observed here should not cause any problems in applying MAb 29B3 for detection of SLCMV in cassava plants. Cross-reactivity with the recombinant CP of ICMV, the other cassava mosaic virus reported to cause CMD in Asia suggested that the developed TAS-ELISA might also be useful for ICMV screening in cassava as well. Nevertheless, since ICMV infecting cassava has never been reported in Thailand to date, we are not able to perform the cross-reactivity test against ICMV in infected plants at the moment.

The use of disease-free or clean cassava stems as planting materials has been considered a key step to reduce yield loss and the incidence of CMD. In this study, we investigated whether the newly developed TAS-ELISA can be applied for screening of cassava stems. Since cassava stems usually do not bear leaves, we therefore developed protocols for both direct and indirect detection of SLCMV from cassava stems. For direct detection, green barks from cassava stem tips were chosen as representative samples based on our previous finding that they yielded more accurate and consistent results for SLCMV detection by both TAS-ELISA and PCR (compared to the other parts of cassava stems, e.g. root, bud, brown bark). However, since most cassava planting stems do not contain the green part, we therefore developed a protocol for indirect detection of SLCMV using young leaf sprouts from cassava cuttings after being grown in a greenhouse for 7–14 days. SLCMV detection using young leaves actively sprouting from cassava cuttings can minimize the difficulties regarding virus detection at low titers usually associated with the ELISA platform. Our results showed that, besides young leaves obtained from stem cuttings of symptomatic cassava plants, our method was also able to detect SLCMV in some of the young leaf samples obtained from stem cuttings of cassava plants without CMD symptoms. It should also be noted that, while it usually takes several weeks before CMD-affected young leaves can be distinguished visually, our TAS-ELISA system can detect SLCMV in leaf sprouts as early as 7–10 days (usually 0.5–2 cm in size) after planting.

In term of surveillance, since CMD-free cuttings can still be infected by viruliferous whiteflies after planting, field inspection should therefore be performed regularly so that newly infected plants which serve as reservoirs for CMD can be identified and removed from the field as soon as possible. Our studies in field-collected leaf samples demonstrated that, besides all symptomatic leaves, TAS-ELISA was also able to detect SLCMV in symptomless cassava leaf samples. Even though TAS-ELISA gave 2 false negative results in field-collected cassava leaves this study, it demonstrated an overall high accuracy rate compared to PCR. It should also be noted that the relative sensitivity of the newly developed TAS-ELISA for SLCMV detection is slightly different among the types of cassava tissues: 96.7% for field-collected leaves (n = 114) and 100% for young leaf sprouts (n = 352) and green barks (n = 17) from cassava stems.

In addition to cassava plants, our developed TAS-ELISA also allows detection of SLCMV from chaya (*Cnidoscolus aconitifolius*) and coral plants (*Jatropha multifida*), both of which belong to the same family as cassava (*Euphorbiaceae*) and can be propagated through stem cuttings. SLCMV infecting chaya was reported previously in India [33], whereas the two CMBs previously reported in coral plant were *African cassava mosaic virus* (ACMV) and *East African cassava mosaic virus* (EACMV) [17, 34]. To our knowledge, this is the first report of SLCMV in plants other than cassava in Thailand and SEA. Nevertheless, whether the presence of SLCMV in these plants plays any role in the spread and maintenance of CMD needs to be studied further.

## Conclusions

In conclusion, an MAb to SLCMV was generated and successfully used to develop TAS-ELISA for SLCMV detection. The newly developed TAS-ELISA using PAb PK and MAb 29B3 has a high overall accuracy rate (99.6%) for SLCMV detection in field-collected cassava leaves, young leaf sprouts of cassava stem cuttings, and green barks from cassava stem tips as compared to the reference PCR. Our findings suggest that our TAS-ELISA assay can serve as an attractive tool for inexpensive and high-throughput detection of SLCMV and can have implications for CMD screening of cassava stem cuttings, large-scale surveillance, and screening for resistance.

## Supplementary Information

**Additional file 1. Table S1**: List of primers used in this study.

**Additional file 2. Fig. S1**: Agarose gel electrophoreses showing (**A**) SLCMV detection by PCR using SLCMV-specific primers, and (**B**) ICMV detection by PCR using ICMV-specific primers. Lane M: 100 bp DNA ladder (Thermo Fisher Scientific, USA); Lane 1: SLCMV-infected cassava from Sisaket province [SSK3-14]; Lane 2: SLCMV-infected cassava from Prachin Buri province [PRJ-44]; Lane 3: healthy cassava; Lane 4: ICMV positive control (pUC-Amp carrying a synthesized ICMV DNA fragment covering the ICMV DNA-A genome (AJ314739) from positions 1664 to 2463) (Integrated DNA Technologies, Inc, USA); Lane 5: SLCMV positive control (DSMZ, Germany); Lane 6: distilled water. Arrows indicate the size of target PCR product (616 bp for SLCMV-specific primers and 713 bp for ICMV-specific primers). (See Additional file 1: Table S1 for primer details).

**Additional file 3. Fig. S2**: Agarose gel electrophoresis showing representative results of SLCMV detection in field-collected samples by PCR using SLCMV-specific primers Lane M: 1 kb DNA ladder (Thermo Fisher Scientific, USA); Lane 1–28: field-collected cassava leaf samples; DW: distilled water; SLCMV PC: SLCMV positive control. Arrow indicates the size of target PCR product (616-bp).

**Additional file 4. Table S2**: Relative accuracy, relative specificity, and relative sensitivity of the developed TAS-ELISA as compared to the PCR.

## Data Availability

All data generated or analyzed during this study are included in this published article and its additional files. Nucleotide sequences of the full-length SLCMV-CP gene derived from SLCMV-infected cassava leaf tissue (SLCMV-[SSK3-14]) as well as partial Rep gene sequences derived from SLCMV-infected chaya (SLCMV-[ChY-1]) and coral plants (SLCMV-[CP-870]) are available at http://www.ncbi.nlm.nih.gov/genbank/. The accession numbers are as follows. SLCMV-[SSK3-14], MN970012; SLCMV-[ChY-1], MT921843 and SLCMV-[CP-870], MT921844.

## Abbreviations

CMD: Cassava mosaic disease
CMBs: Cassava mosaic begomoviruses
SLCMV: *Sri Lankan cassava mosaic virus*
ICMV: *Indian cassava mosaic virus*
SEA: Southeast Asia
PCR: Polymerase chain reaction
DAS-ELISA: Double antibody sandwich enzyme-linked immunosorbent assay
TAS-ELISA: Triple antibody sandwich enzyme-linked immunosorbent assay
RCA: Rolling circle amplification
PTA-ELISA: Plate-trapped antigen enzyme-linked immunosorbent assay
MAbs: Monoclonal antibodies
CP: Coat protein
qPCR: Quantitative real-time PCR
AYVV: *Ageratum yellow vein virus*
PepYLCIV: *Pepper yellow leaf curl Indonesia virus*
PepYLCTHV: *Pepper yellow leaf curl Thailand virus*
SLCCNV: *Squash leaf curl China virus*
ToLCNDV: *Tomato leaf curl New Delhi virus*
TYLCKaV: *Tomato yellow leaf curl Kanchanaburi virus*
TYLCTHV: *Tomato yellow leaf curl Thailand virus*
PAb: Polyclonal antibody
ACMV: *African cassava mosaic virus*
EACMV: *East African cassava mosaic virus*
Cq: Quantification cycle

## References

[1] Food and Agriculture Organization of the United Nations. FAOSTAT statistics database, FAO (2018). http://www.fao.org/faostat/en/#data/QC. Accessed 22 Nov 2020.

[2] Sarker MNI, Hossin MA, Yin X, Sarkar MK (2018). One belt one road initiative of China: implication for future of global development. Mod Econ.

[3] Thai Tapioca Starch Association (TTSA). Export tapioca products (2019). http://www.thaitapiocastarch.org/en/information/statistics/export_tapioca_products. Accessed 22 Nov 2020.

[4] Patil BL, Fauquet CM (2009). Cassava mosaic geminiviruses: actual knowledge and perspectives. Mol Plant Pathol.

[5] Rybicki EP (2015). A top ten list for economically important plant viruses. Arch Virol.

[6] Rey C, Vanderschuren H (2017). Cassava mosaic and brown streak diseases: current perspectives and beyond. Annu Rev Virol.

[7] Hong YG, Robinson DJ, Harrison BD (1993). Nucleotide sequence evidence for occurrence of three distinct whitefly-transmitted geminiviruses in cassava. J Gen Virol.

[8] Saunders K, Salim N, Mali VR, Malathi VG, Briddon R, Markham PG, Stanley J (2002). Characterisation of *Sri Lankan cassava mosaic virus* and *Indian cassava mosaic virus*: evidence for acquisition of a DNA B component by a monopartite begomovirus. Virology.

[9] Dutt N, Briddon RW, Dasgupta I (2005). Identification of a second begomovirus, Sri Lankan cassava mosaic virus, causing cassava mosaic disease in India. Arch Virol.

[10] Jose A, Makeshkumar T, Edison S (2011). Survey of cassava mosaic disease in Kerala. J Root Crops.

[11] Wang HL, Cui XY, Wang XW, Liu SS, Zhang ZH, Zhou XP (2016). First report of *Sri Lankan cassava mosaic virus* infecting cassava in Cambodia. Plant Dis.

[12] Uke A, Hoat TX, Quan MV, Liem NV, Ugaki M, Natsuaki KT (2018). First report of *Sri Lankan cassava mosaic virus* infecting cassava in Vietnam. Plant Dis.

[13] Siriwan W, Jimenez J, Hemniam N, Saokham K, Lopez-Alvarez D, Leiva AM, Martinez A, Mwanzia L, Lopez-Lavalle LAB, Cuellar WJ (2020). Surveillance and diagnostics of the emergent Sri *Lankan cassava mosaic virus* (Fam. *Geminiviridae*) in Southeast Asia. Virus Res..

[14] Wang D, Yao XM, Huang GX, Shi T, Wang GF, Ye J (2019). First report of *Sri Lankan cassava mosaic virus* infected cassava in China. Plant Dis.

[15] Wang D, Huang GX, Shi T, Wang GF, Fang RX, Zhang X, Ye J (2020). Surveillance and distribution of the emergent Sri Lankan cassava mosaic virus in China. Phytopathol Res.

[16] Bock KR, Woods RD (1983). Etiology of *African cassava mosaic disease*. Plant Dis.

[17] Fauquet C (1990). Fargette D *African cassava mosaic virus*: etiology, epidemiology and control. Plant Dis.

[18] Thresh JM, Fargette D, Otim-Nape GW (1994). Effects of African cassava mosaic geminivirus on the yield of cassava. Trop Sci Lond.

[19] Legg JP, Kumar PL, Makeshkumar T, Tripathi L, Ferguson M, Kanju E, Ntawuruhunga P, Cuellar W (2015). Cassava virus diseases: biology, epidemiology, and management. Adv Virus Res.

[20] Makeshkumar T, Sankar A, Nair RR, Edison S (2005). Detection of Cassava mosaic virus in India: using polymerase chain reaction and nucleic acid hybridization technique. J Root Crops.

[21] Aloyce RC, Tairo F, Sseruwagi P, Rey MEC, Ndunguru J (2013). A single-tube duplex and multiplex PCR for simultaneous detection of four cassava mosaic begomovirus species in cassava plants. J Virol Methods.

[22] Naseem S, Winter S (2016). Quantification of *African cassava mosaic virus* (ACMV) and *East African cassava mosaic virus* (EACMV-UG) in single and mixed infected cassava (*Manihot esculenta* Crantz) using quantitative PCR. J Virol Methods.

[23] Amoatey HM, Appiah AS, Danso KE, Amiteye S, Appiah R, Klu GYP, Owusu GK (2013). Controlled transmission of *African cassava mosaic virus* (ACMV) by *Bemisia tabaci* from cassava (*Manihot esculenta* Crantz) to seedlings of physic nut (*Jatropha curcas* L.). Afr J Biotechnol.

[24] Givord L, Fargette D, Kounounguissa B, Thouvenel JC, Walter B, Van Regenmortel MHV (1994). Detection of geminiviruses from tropical countries by a double monoclonal antibody ELISA using antibodies to *African cassava mosaic virus*. Agronomie.

[25] Iwalaye OA, Ala AA (2015). Production of *African cassava mosaic virus* (ACMV) specific polyclonal antibody by oral immunization of mice. IOSR J Pharm Biol Sci.

[26] Karthikeyan C, Patil BL, Borah BK, Resmi TR, Turco S, Pooggin MM, Hohn T, Veluthambi K (2016). Emergence of a latent *Indian cassava mosaic virus* from cassava which recovered from infection by a non-persistent *Sri Lankan cassava mosaic virus*. Viruses.

[27] Jose A, Makeshkumar T, Edison S (2008). Host range of Sri Lankan cassava mosaic virus. J Root Crops.

[28] Minato N, Sok S, Chen S, Delaquis E, Phirun I, Le VX, Burra DD, Newby JC, Wyckhuys KAG, de Haan S (2019). Surveillance for *Sri Lankan cassava mosaic virus* (SLCMV) in Cambodia and Vietnam one year after its initial detection in a single plantation in 2015. PLoS ONE.

[29] Duraisamy R, Natesan S, Muthurajan R, Gandhi K, Lakshmanan P, Karuppusamy N, Chokkappan M (2013). Molecular studies on the transmission of *Indian cassava mosaic virus* (ICMV) and *Sri Lankan cassava mosaic virus* (SLCMV) in cassava by *Bemisia tabaci* and cloning of ICMV and SLCMV replicase gene from cassava. Mol Biotechnol.

[30] Ntui VO, Kong K, Khan RS, Igawa T, Janavi GJ, Rabindran R, Nakamura I, Mii M (2015). Resistance to *Sri Lankan cassava mosaic virus* (SLCMV) in genetically engineered cassava cv. KU50 through RNA silencing. PLoS ONE.

[31] Seepiban C, Charoenvilaisiri S, Warin N, Bhunchoth A, Phironrit N, Phuangrat B, Chatchawankanphanich O, Attathom S, Gajanandana O (2017). Development and application of triple antibody sandwich enzyme-linked immunosorbent assays for begomovirus detection using monoclonal antibodies against *Tomato yellow leaf curl Thailand virus*. Virol J.

[32] Banoo S, Bell D, Bossuyt P, Herring A, Mabey D, Poole F, Smith PG, Sriram N, Wongsrichanalai C, Linke R, O'Brien R, Perkins M, Cunningham J, Matsoso P, Nathanson CM, Olliaro P, Peeling RW, Ramsay A (2006). Evaluation of diagnostic tests for infectious diseases: general principles. Nat Rev Microbiol.

[33] Snehi SK, Purvia AS, Gupta G, Parihar SS, Singh V (2017). Molecular detection of a begomovirus species on chaya (*Cnidoscolus acontifolia*) from Madhya Pradesh, India which is distantly related to *Sri Lankan cassava mosaic virus*. Virol Mycol.

[34] Ramkat R, Calari A, Maghuly F, Laimer M (2011). Occurrence of *African cassava mosaic virus* (ACMV) and *East African cassava mosaic virus*–Uganda (EACMV-UG) in *Jatropha curcas*. BMC Proc.

[35] Harrison BD, Swanson MM, Fargette D (2002). Begomovirus coat protein: serology, variation and functions. Physiol Mol Plant Pathol.

[36] Kumar RV (2019). Plant antiviral immunity against geminiviruses and viral counter-defense for survival. Front Microbiol.

