# Supplementary material for: Development of a triple antibody sandwich enzyme-linked immunosorbent assay for cassava mosaic disease detection using a monoclonal antibody to Sri Lankan cassava mosaic virus
Source: Virol J. 2021 May 18;18:100. doi: 10.1186/s12985-021-01572-6 (PMC8130424; doi:10.1186/s12985-021-01572-6)
Supplement: Supplementary file 1 — Additional file 1. Table S1: List of primers used in this study. [file 12985_2021_1572_MOESM1_ESM.docx]

**Additional file 1: Table S1** List of primers used in this study.

| Primer name | Nucleotide sequence (5’-3’) | Position ^a^ | Size^b^ | Purpose | Reference |
| --- | --- | --- | --- | --- | --- |
| SLCMV-F | GAA GGG AGA CAC ATA TAC CTC G | 2054-2075 | 616 | SLCMV detection | [26] |
| SLCMV-R | CAC ATA TAT ATT GTC TCC AAT TCA C | 2669-2645 |  |  |  |
| SLCMV-CPF | GGA TCC ATG TCG AAG CGA CCA GCA | 298-351 | 771 | Cloning of SLCMV CP gene | This study |
| SLCMV-CPR | AAG CTT TTA ATT GCT CAC TGA ATC | 1068-1051 |  |  |  |
| SLCMV2.5-F | GCA AGG AAC AGG CTT TAG T | 875-893 | 2521 | Cloning of 2.5-kb  SLCMV DNA-A fragment |  |
| SLCMV2.5-R | GGA CTT AAC GCA AAA CCT CT | 636-617 |  |  |  |
| SLCMV-Q1F2378 | GAA TTG CCG ATT GTT TGT GAT TGT G | 2378-2402 | 136 | Quantification of SLCMV copy no. by qPCR |  |
| SLCMV-Q1R2197 | GCA CTT AAC ACA GGC AGT AAG C | 2197-2176 |  |  |  |
| Beg-F 1692 | GGA TTG CAG AGG AAG ATA GTG GG | 1692-1714 | 713 | ICMV detection |  |
| ICMV-R2404 | TAC AAA TGC CAG AAT CAG CG | 2404-2385 |  |  |  |

^a^ Position based on GenBank accession no. MN544647 for SLCMV primers, and GenBank accession no. AJ314739 for ICMV primers.

^b^ Amplicon size (bp)
