# Supplementary material for: Development of a triple antibody sandwich enzyme-linked immunosorbent assay for cassava mosaic disease detection using a monoclonal antibody to Sri Lankan cassava mosaic virus
Source: Virol J. 2021 May 18;18:100. doi: 10.1186/s12985-021-01572-6 (PMC8130424; doi:10.1186/s12985-021-01572-6)
Supplement: Supplementary file 2 — Additional file 2. Fig. S1: Agarose gel electrophoreses showing (A) SLCMV detection by PCR using SLCMV-specific primers, and (B) ICMV detection by PCR using ICMV-specific primers. Lane M: 100 bp DNA ladder (Thermo Fisher Scientific, USA); Lane 1: SLCMV-infected cassava from Sisaket province [SSK3-14]; Lane 2: SLCMV-infected cassava from Prachin Buri province [PRJ-44]; Lane 3: healthy cassava; Lane 4: ICMV positive control (pUC-Amp carrying a synthesized ICMV DNA fragment covering the ICMV DNA-A genome (AJ314739) from positions 1664 to 2463) (Integrated DNA Technologies, Inc, USA); Lane 5: SLCMV positive control (DSMZ, Germany); Lane 6: distilled water. Arrows indicate the size of target PCR product (616 bp for SLCMV-specific primers and 713 bp for ICMV-specific primers). (See Additional file 1: Table S1 for primer details). [file 12985_2021_1572_MOESM2_ESM.docx]

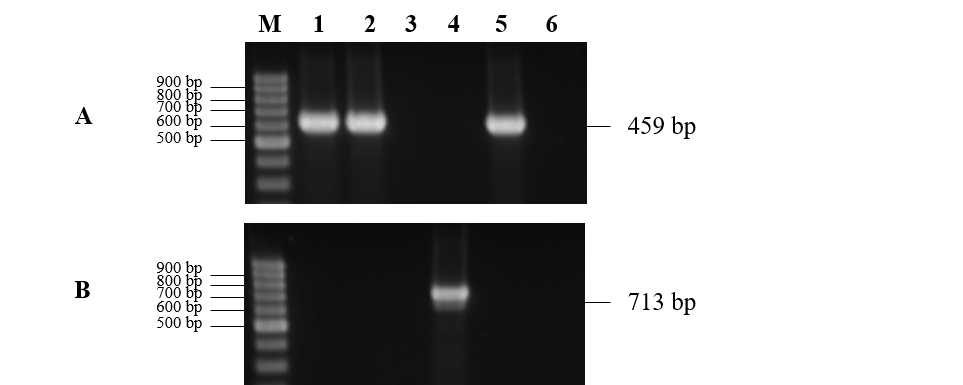


**Additional file 2 Fig. S1.** Agarose gel electrophoreses showing (**A**) SLCMV detection by PCR using SLCMV-specific primers, and (**B**) ICMV detection by PCR using ICMV-specific primers. Lane M: 100 bp DNA ladder (Thermo Fisher Scientific, USA); Lane 1: SLCMV-infected cassava from Sisaket province [SSK3-14]; Lane 2: SLCMV-infected cassava from Prachin Buri province [PRJ-44]; Lane 3: healthy cassava; Lane 4: ICMV positive control (pUC-Amp carrying a synthesized ICMV DNA fragment covering the ICMV DNA-A genome (AJ314739) from positions 1664 to 2463) (Integrated DNA Technologies, Inc, USA); Lane 5: SLCMV positive control (DSMZ, Germany); Lane 6: distilled water. Arrows indicate the size of target PCR product (616 bp for SLCMV-specific primers and 713 bp for ICMV-specific primers). (See Additional file 1: Table S1 for primer details).
