# Supplementary material for: Development of a triple antibody sandwich enzyme-linked immunosorbent assay for cassava mosaic disease detection using a monoclonal antibody to Sri Lankan cassava mosaic virus
Source: Virol J. 2021 May 18;18:100. doi: 10.1186/s12985-021-01572-6 (PMC8130424; doi:10.1186/s12985-021-01572-6)
Supplement: Supplementary file 3 — Additional file 3. Fig. S2: Agarose gel electrophoresis showing representative results of SLCMV detection in field-collected samples by PCR using SLCMV-specific primers Lane M: 1 kb DNA ladder (Thermo Fisher Scientific, USA); Lane 1–28: field-collected cassava leaf samples; DW: distilled water; SLCMV PC: SLCMV positive control. Arrow indicates the size of target PCR product (616-bp). [file 12985_2021_1572_MOESM3_ESM.docx]

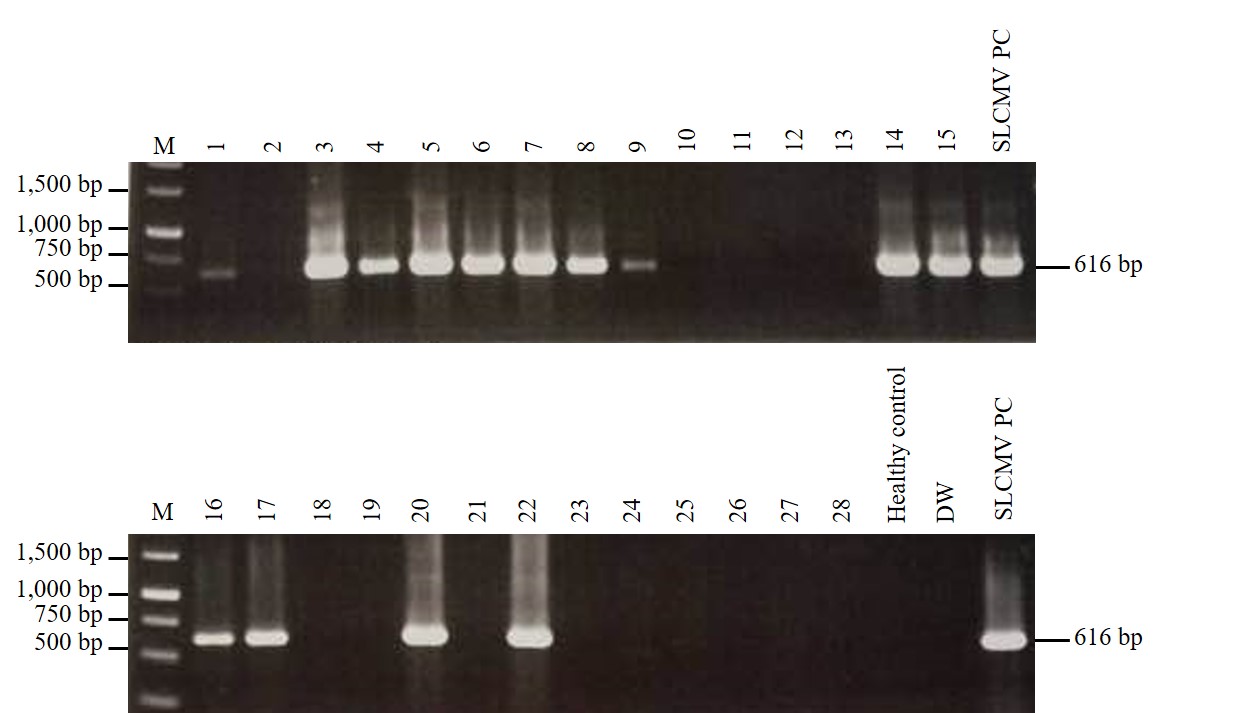


**Additional file 3 Fig. S2. Agarose gel electrophoreses showing representative results of SLCMV detection in field-collected samples by PCR using SLCMV-specific primers.** Lane M: 1 kb DNA ladder (Thermo Fisher Scientific, USA); Lane 1-28: field-collected cassava leaf samples; DW: distilled water; SLCMV PC: SLCMV positive control. Arrow indicates the size of target PCR product (616-bp).
