# Supplementary material for: Development of a triple antibody sandwich enzyme-linked immunosorbent assay for cassava mosaic disease detection using a monoclonal antibody to Sri Lankan cassava mosaic virus
Source: Virol J. 2021 May 18;18:100. doi: 10.1186/s12985-021-01572-6 (PMC8130424; doi:10.1186/s12985-021-01572-6)
Supplement: Supplementary file 4 — Additional file 4. Table S2: Relative accuracy, relative specificity, and relative sensitivity of the developed TAS-ELISA as compared to the PCR. [file 12985_2021_1572_MOESM4_ESM.docx]

**Additional file 4: Table S2.** Relative accuracy, relative specificity, and relative sensitivity of the developed TAS-ELISA as compared to the PCR

| Sample types | Number^a^ | | | | Accuracy  % | Specificity  % | Sensitivity  % |
| --- | --- | --- | --- | --- | --- | --- | --- |
|  | PD | PA | NA | ND |  |  |  |
| Field-collected cassava leaf samples |  |  |  |  |  |  |  |
| Leaves with CMD symptoms | 0 | 57 | 0 | 0 |  |  |  |
| Leaves without CMD symptoms | 0 | 2 | 53 | 2 |  |  |  |
| Total | 0 | 59 | 53 | 2 | 98.2 | 100 | 96.7 |
| Young leaf sprouts from cassava stem cuttings |  |  |  |  |  |  |  |
| Stem cuttings from cassava with CMD symptoms | 0 | 35 | 0 | 0 |  |  |  |
| Stem cuttings from cassava without CMD symptoms | 0 | 4 | 38 | 0 |  |  |  |
| Stem cuttings randomly purchased from local farmers | 0 | 0 | 275 | 0 |  |  |  |
| Total | 0 | 39 | 313 | 0 | 100 | 100 | 100 |
| Green bark at the tip of cassava stems |  |  |  |  |  |  |  |
| Stem cuttings from cassava with CMD symptoms | 0 | 5 | 0 | 0 |  |  |  |
| Stem cuttings from cassava without CMD symptoms | 0 | 1 | 11 | 0 |  |  |  |
| Total | 0 | 6 | 11 | 0 | 100 | 100 | 100 |
| Other plants |  |  |  |  |  |  |  |
| Chaya leaves with CMD-like symptoms | 0 | 3 | 0 | 0 |  |  |  |
| Chaya leaves without CMD-like symptoms | 0 | 0 | 3 | 0 |  |  |  |
| Coral plant leaves with CMD-like symptoms | 0 | 1 | 0 | 0 |  |  |  |
| Total | 0 | 4 | 3 | 0 | 100 | 100 | 100 |
| **Total samples** | **0** | **108** | **380** | **2** | **99.6** | **100** | **98.2** |

^a^ Results obtained by TAS-ELISA were compared with those obtained by PCR, and samples were defined as positive agreement (PA), negative agreement (NA), positive deviation (PD) and negative deviation (ND). Relative accuracy, specificity and sensitivity were then calculated as follows: Relative accuracy = (PA+NA)/N × 100%; relative specificity = (NA/ (PD+NA)) × 100%; relative sensitivity = (PA/ (PA + ND)) × 100%, where N is the total number of samples (PA + NA + PD + ND).
